# Parkinson's patients in the Brazilian Public Health Policy context

**DOI:** 10.1590/S1679-45082016ED3780

**Published:** 2016

**Affiliations:** 1 Hospital Israelita Albert Einstein, São Paulo, SP, Brazil

Demographic profiles are changing in Brazil and worldwide. If, in the 1950's, we saw a pyramid represented in age group graphs, the trend for 2060 is that we will probably see a rectangle.^(1)^ This means we are aging, and individuals aged over 60 years, in Brazil, will account for approximately 33.7% of the entire population.^(2)^ That is an alarming number for society as a whole, especially for Public Health managers who need new strategies to cope with the impact this will bring to everyone.

We need to have detailed knowledge of demographic, social, cultural, economic and health-related aspects, among others, when referring to certain population segments, such as in the case of seniors. They acquire representation in societies, and this must be translated as the main foundation for the establishment of policies to meet the demands of these contingents, whether these policies are public or private,^(3)^ because aging is a process linked to all of society, and it should not be met with discrimination.

With this new reality, it is expected that diseases that are characteristic of seniors start to be more prevalent. That is the case with Parkinson's disease (PD), the second most prevalent neurodegenerative disease with long survival time in the world. Parkinson's disease often affects individuals still in their productive phase, generally at 40 to 50 years old, compromising their quality of life and aging, and it is one of the most expensive neurological diseases of old age.^(4,5)^

In Brazil, reporting of PD is not compulsory, which results in a merely estimated number of its prevalence in Brazil. It is estimated as 220 thousand patients, and there are international studies that suggest this number will increase by more than double that by 2030.^(6)^ However, if we take into account that, in 2009, the country had an over-60 population of approximately 21 million people.^(7)^ According to a research carried out in a city in the interior of the state of Minas Gerais (MG), Parkison's patients accounted for 3.3% of this over-60 population,^(8)^ which means over 630,000 people suffering from PD.

The numbers are alarming due to the huge health care charges the disease brings. Parkinson's disease patients are the ones who most use health services and need medication for the rest of their lives, they are more likely to be admitted to hospitals due to their disease or other correlated factors, and need home care and home adaptations for their convenience and safety. Considering the individual may still be in his/her productive phase, the cost they bring to society may last a long time. Life expectancy for 2030, *e.g.*, is estimated at 78.6 years and, for 2060, that number rises to 81.2 years.^(9)^ Add that to the current financial crisis, which “forces” people into the Brazilian public healthcare system (SUS -*Sistema Único de Saúde*) who can no longer afford private health insurance plans, thus increasing their usual demand for health care.

Actions by the State, in regards to the population's quality of life, are done through Public Policies, which are guidelines for public power actions, generally involving public resources, and are written as documents (laws, programs, credit lines).^(10,11)^ Among these Public Policies, are Health Policies, devoted to social protection.

The Ministry of Health, through the Secretariat of Health Care, published an ordinance (ordinance 228, of May 10^th^, 2010)^(12)^ designed especially to establish parameters about PD in Brazil and its guidelines for diagnosis, treatment and monitoring of individuals with the disease. This ordinance states the importance of the disease in the Brazilian (elderly) population by the Federal Government, as one of the legal frameworks for the orientation of actions in the field of aging, included in Clinical Protocols and Therapeutic Guidelines.^(13)^

Many benefits and rights are ensured to Parkinson's patients (as well as to other special needs individuals) and are often not known by the patients and/or their families and healthcare professionals responsible for them and who may help them improve their condition and quality of life. We can cite a few, such as the dispensing of medications for the disease or other comorbidities related to it; benefits due to disability and, later on, retirement by social security whose total amount may be increased by 25% should the ensured need permanent third-party assistance, which depends on verification through medical expertise of the National Social Security Institute (INSS -*Instituto Nacional de Seguridade Social*); social protection in the amount of one minimum wage, provided the monthly family income (*per capita*) is less than 25% of the minimum wage; exemption from taxes over income that is related to retirement or pension (other forms of income are not exempt), including any complement payment received from a private entity and alimony/child support received by PD patients; exemption from the car license plate rotation instituted in the city of São Paulo (SP), to PD patients who are residents of the capital city. Patients who prove an income at minimum wage can travel between states by bus, train or boat without paying for intermunicipal tickets. They also rely on an exemption of up to 30% over taxes when purchasing new or used vehicles.^(14–16)^

Parkinson's patients can also use all benefits ensured by the Brazilian Law of Inclusion of People with Disabilities,^(17)^ as long as an assessment of disability is performed by a multidisciplinary and interdisciplinary team whenever necessary.

Even though the abovementioned actions are recent and belong to government programs aiming to enable the proposed objectives, and many of these actions are in fact concrete, we have observed that some accomplishments happen due to initiatives by philanthropic associations, religious and/or non-governmental organizations. Even relying on the support from the patients, their family and friends, these action are limited and aid only a small percentage of these patients.

In this context, we highlight more than 30 nongovernmental associations spread over the country that work towards the interests and assistance of Parkinson's patients who, in most cases, rely on their own resources as well as sponsorships from private companies, donations or private actions that ensure their subsistence.

We then need to be mindful not only of quantitative aspects this demographic transition presents us with, but also of qualitative aspects; and the patient comes first. We are living longer, due to changes in the lives of the population in social, economic and cultural areas, and that requires new vision and planning for the substantial improvement of health parameters. However, living longer is not enough. We must add quality to our additional years.

Nowadays, aging is no longer a privilege; it is a reality. And, even if aging with no chronic diseases is an exception, having a disease does not necessarily mean social exclusion.^(7)^ Therefore, let's actualize the existing Public Policies so that the elderly, even with chronic diseases, such as PD, can justly and democratically reach the equity established in the law, thus having a better quality of life to preserve their autonomy for longer, and, in turn, decreasing their cost to the State and the society.
